# Dataset on water–glycerol flow in a horizontal pipeline with and without leaks

**DOI:** 10.1016/j.dib.2020.105950

**Published:** 2020-07-04

**Authors:** José F. Noguera-Polania, J. Hernández-García, Diego F. Galaviz-López, Lizeth Torres, J.E.V. Guzmán, Marco E. Sanjuán-Mejía, Javier Jiménez-Cabas

**Affiliations:** a Departamento de Ingeniería Mecánica, Universidad del Norte, Barranquilla, Colombia; b Instituto de Ingeniería, Universidad Nacional Autónoma de México, Ciudad de México, México; c Departamento de Ciencias de la Computación y Electrónica, Universidad de la Costa, Barranquilla, Colombia

**Keywords:** High-viscosity fluids, Extra-heavy oils, Pipelines, Model validation, Leak detection

## Abstract

The dataset presented in this article was collected in a laboratory flow circuit, which was designed to investigate high-viscosity flows. The data set is composed of 1200 s (equivalent to 12,000 samples) of mass flow and pressure measurements taken at five points along the pipeline. The first 300 s were recorded when the flow in the loop was composed only of glycerol. The remaining data were acquired when the flow was composed of a water–glycerol mixture. During the data acquisition, two extractions were produced. The research reported in [1] uses 160 s of the data provided here. This article explains in detail the experimental set-up and the principal instruments used for obtaining the dataset. The dataset is in the form of seven columns: Time, Mass Flow, Pressure 1, Pressure 2, Pressure 3, Pressure 4, Pressure 5, in supplementary Excel and Matlab files.

Specifications table

**Table:** 

Subject	Mechanical Engineering
Specific subject area	Fluid mechanics
Type of data	Excel files Matlab files
How data were acquired	20 min of measurements (100 ms sampling time) per data using: (1) Mass Flow Transducer (Endress-Hauser Coriolis mass flowmeter, Promass 83F80DN80 3, ± 0.1% Full-Scale error) (5) Pressure Transducers (MEAS U5300, ±1% error)
Data format	Raw
Parameters for data collection	Before any preliminary test, electrical wires connections were checked.
Description of data collection	A progressive cavity pump provides the energy necessary to recirculate the liquid through the pipeline. The inlet mass flow is measured, as well as the pressure at five different points along the pipeline.
Data source location	Institution: Universidad Nacional Autónoma de México City: Mexico City Country: Mexico
Data accessibility	With the article
Related research article	Noguera, J. F., Torres, L., Verde, C., Guzmán, E., Sanjuan, M. Model for the flow of a water-glycerol mixture in horizontal pipelines. 2019 4th Conference on Control and Fault Tolerant Systems (SysTol) (pp. 117–122). IEEExplore. DOI: https://doi.org/10.1109/SYSTOL.2019.8864781

## Value of the data

• The similarity in viscosity of glycerol and extra-heavy crude allows researchers to use the data provided here to evaluate models that describe the flow of extra-heavy crude.
• The glycerol diluted with water emulates the mixture of extra-heavy oils with drag reducing agents (DRA), which are usually employed to reduce the pressure during the transport of extra heavy oils. For this reason, the data provided here can help to researchers and practicioners to evaluate prediction models involving DRA.
• A valve is used to emulate the appearance of leaks. Hence, the data set can be used to validate leak diagnosis approaches similar to [2].
• The dataset can benefit pipeline owners, operators, and researchers since this can be used not only for validating pipeline dynamical models but for improving the transport operation and the accuracy of leak detection systems.
• The data can be used as inputs and outputs to validate models that describe the flow of pure glycerol and the flow of water-glycerol in pressurized horizontal pipes. This can be done only by separating the portions of the time series that were recorded when only glycerol was flowing in the pipeline or when the aqueous glycerol was flowing.
• The pressures taken at the beginning and at the end of the curve section can help analyze the behavior of the glycerol flow when circulating U-shaped sections.

## 1 Data description

**Fig. 1:**
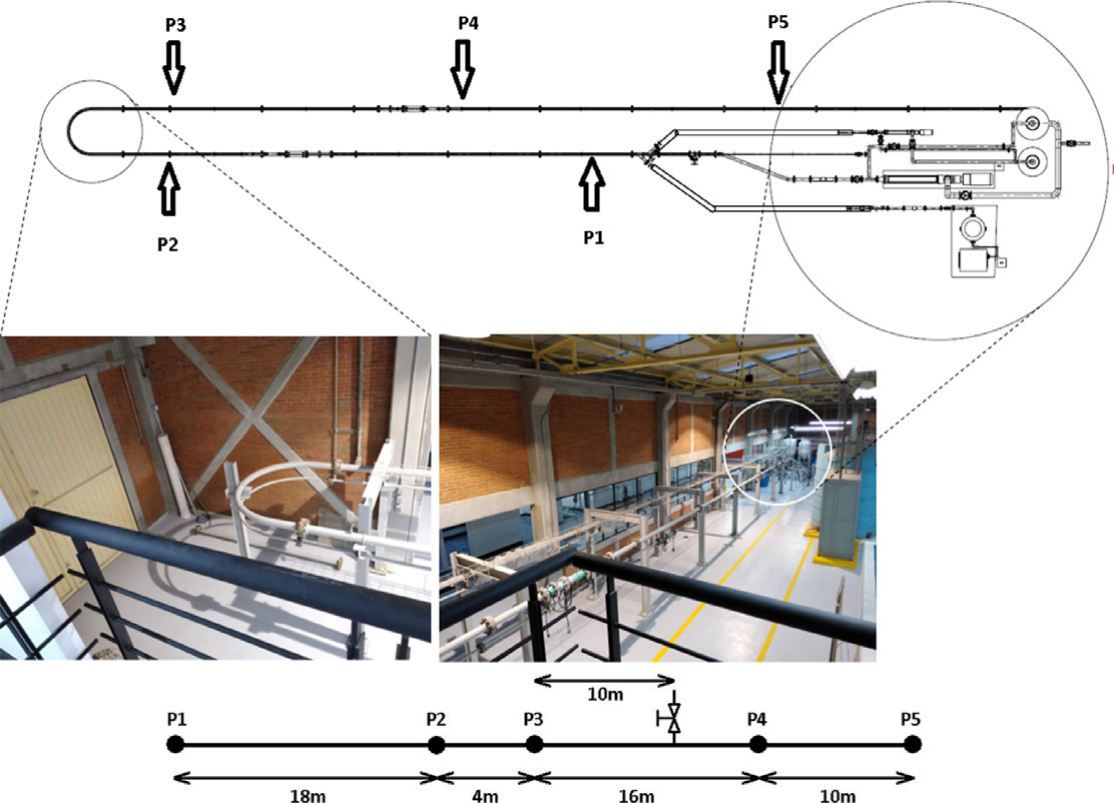
Laboratory flow circuit.

This paper presents the experimental dataset obtained from a laboratory flow circuit located at Instituto de Ingeniería of Universidad Nacional Autónoma de México. The prototype in Fig. 1, is equipped with: a steel pipeline of 0.0762 m (3 in) of diameter and 54 m of length, a 40HP progressive cavity pump (Seepex Mod. BN35-24), five pressure measurement intermediate points (P1 to P5), a mass flow sensor installed at the inlet of the pipeline and a 1.5 m^3^ separator tank. These measurements are shared in a supplementary file in Excel and Matlab format.

## 2 Experimental design, materials and methods

Fig. 1 shows the pipeline utilized to perform experimental investigations. In the experiment, the viscosity of pure glycerol was 0.460 Pa.s, and the viscosity of diluted glycerol was 0.007 Pa.s. The experiment process is described here below.

• The progressive cavity pump turns on at time *t* = 63.37 s, and it reaches a mean mass flow of 6.26 kg/s of pure glycerol.
• Glycerol diluted with water was injected at time *t* = 321 s, in a proportion of 45% glycerol and 55% water. The mass flow of diluted glycerol injected was 5% of the mass flow of pure glycerin.
• At time *t* = 567 s, a leak, with a mass flow Q_l_ = 0.0126 kg/s, was induced by opening a valve located 10 m downstream from pressure sensor P3 (Fig. 1). This valve was closed at time *t* = 668 s.
• At time *t* = 779 s, the diluted glycerol injection was stopped.
• At time *t* = 1045s a second leak, with a mass flow Q_l_ = 0.0127 kg/s, was induced. In this case, the valve was closed at time *t* = 1136s.

**Fig. 2:**
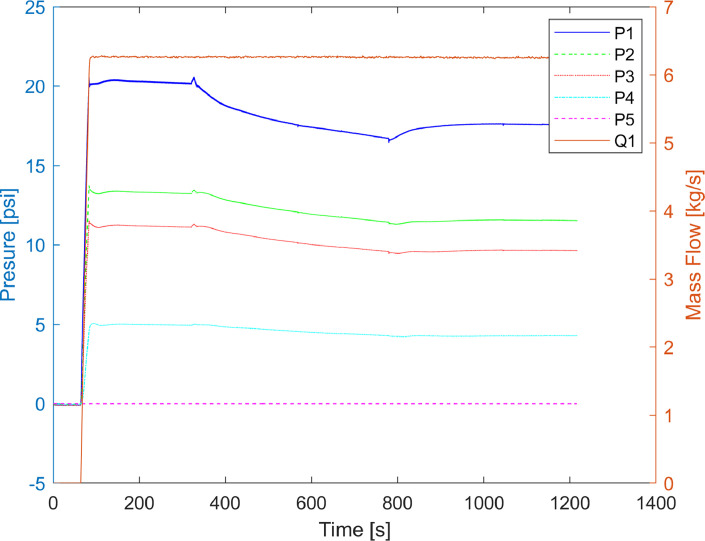
Laboratory pipeline mass flow and pressures.

In Fig. 2 are shown the measurements of the five pressure sensors (P1 to P5) and the mass flow sensor at the pipeline inlet (Q1). Also, the Matlab file to plot the figure is shared

## Declaration of Competing Interest

The authors declare that they have no known competing financial interests or personal relationships which have, or could be perceived to have, influenced the work reported in this article.

## Appendix Supplementary materials

Image, application 1

Image, application 2

Image, application 3
